# Evolution of growth strategy in alligators and caimans informed by osteohistology of the late Eocene early‐diverging alligatoroid crocodylian *Diplocynodon hantoniensis*

**DOI:** 10.1111/joa.14231

**Published:** 2025-02-09

**Authors:** D. K. Hoffman, E. R. Goldsmith, A. Houssaye, S. C. R. Maidment, R. N. Felice, P. D. Mannion

**Affiliations:** ^1^ Department of Earth Sciences University College London London UK; ^2^ Department of Geosciences Virginia Tech Blacksburg Virginia USA; ^3^ Département Adaptations du Vivant, UMR 7179 CNRS Muséum National d'Histoire Naturelle Paris France; ^4^ Fossil Reptiles, Amphibians and Birds Section Natural History Museum London London UK; ^5^ School of Geography, Earth and Environmental Sciences University of Birmingham Birmingham UK; ^6^ Science Group Natural History Museum London London UK; ^7^ Centre for Integrative Anatomy, Department of Cell and Developmental Biology University College London London UK

**Keywords:** allometry, histology, microanatomy, ontogeny

## Abstract

Among living crocodylians, alligatoroids exhibit a wide range of body sizes and a biogeographic distribution that spans tropical‐to‐subtropical climates. The fossil record of alligatoroids, however, reveals even greater diversity, including multiple examples of gigantism and a broader distribution that extends into polar latitudes. Osteohistological studies on extant alligatoroids show that living alligators and caimans both exhibit seasonal growth, with roughly comparable growth rates. However, alligators and caimans diverged from one another over 60 million years ago; the dearth of studies on extinct alligatoroids makes it unclear if the shared condition in extant taxa reflects convergent responses to rapid climatic changes in the recent past or represents the ancestral condition in alligatoroids. Additionally, sample sizes are often limited to one or two individuals, especially in extinct crocodylians, obscuring any intraspecific variation present. To address this uncertainty, we conducted the largest monospecific osteohistological study of an extinct crocodylian to date, based on a sample of nine femora, providing unique insight into the intraspecific variation in growth of the early‐diverging alligatoroid *Diplocynodon hantoniensis* from the late Eocene of the UK. The bone microanatomy of *D. hantoniensis* shows moderate compactness, with a well‐defined medullary cavity, and osteohistological features that are generally consistent with those of extant alligatoroids. Samples vary greatly along a continuum in the degree of remodelling and vascularity, highlighting both the importance of evaluating intraspecific variation and limitations of basing histological assessments on singleton samples. Ontogenetic assessment indicates that our sample captures a range of skeletally immature to mature individuals, approximately corresponding to femoral size, but with notable exceptions possibly driven by sexual dimorphism. Body size estimates for *D. hantoniensis* (1.2–3.4 m) fall within the typical range of living American alligators (*Alligator mississippiensis*). Reconstruction from cyclical growth marks indicates a similar overall growth rate between *D. hantoniensis* and *A*. *mississippiensis*. As in extant alligatoroids more generally, this is determinate, seasonally‐controlled growth. Femoral circumference scales positively with femoral length in *D. hantoniensis*, demonstrating similar allometry to *A*. *mississippiensis*. This differs from some other extant crocodylians (e.g. *Crocodylus niloticus* and *Crocodylus johnstoni*) and suggests conservation of allometric relationships in alligatoroids. This in‐depth look into an early diverging alligatoroid indicates that seasonality and growth rates present in extant members were established near the base of the clade. Furthermore, it highlights the importance of including larger samples of singular species in order to capture potential variation when making clade‐wide interpretations.

## Graphical abstract

Living alligatoroids (alligators and caimans) share osteohistological features and display similar overall growth rates. However, it is unclear if these shared patterns are the result of convergent responses or reflect an ancestral condition. In this study, we histologically sampled nine femora of the fossil alligatoroid *Diplocynodon hantoniensis* to better understand growth early in the clade and capture intraspecific variation. Our findings show allometric similarities between *D. hantoniensis* and *Alligator mississippiensis* and indicate growth in living alligatoroids is similar to that of early‐diverging alligatoroids.

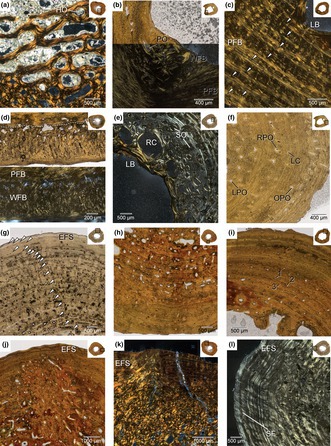

## 1 INTRODUCTION

Today, crocodylians are represented by 28 currently recognised species, characterized by a faunivorous, semi‐aquatic ecology, with a tropical‐to‐subtropical latitudinal distribution (Grigg & Kirshner, 2015; Murray et al., 2019). However, their fossil record reveals a far greater diversity taxonomically, ecomorphologically, and latitudinally (e.g. Mannion et al., 2015; Markwick, 1998; Melstrom & Irmis, 2019; Stubbs et al., 2021; Wilberg et al., 2019). This is perhaps best exemplified by Alligatoroidea (sensu Brochu, 1999), which today is represented by two lineages: Alligatorinae, comprising only two species, the American and Chinese alligators; and Caimaninae, consisting of six species, which are restricted to Central and South America (Grigg & Kirshner, 2015; Roberto et al., 2020). By contrast, more than 40 extinct alligatoroid species are currently recognised since the first appearance of the clade in the fossil record approximately 75 million years ago in the Late Cretaceous (e.g. Brochu, 1999; Cidade et al., 2019; Massonne et al., 2019; Rio et al., 2020; Scheyer et al., 2013; Salas‐Gismondi et al., 2015). Extinct alligatoroids reveal evidence for wide ecomorphological diversity, including ‘duck‐faced’ forms such as *Mourasuchus* (Price, 1964), durophagous species, including *Brachychampsa* (Melstrom & Irmis, 2019; Salas‐Gismondi et al., 2015), and giants such as *Deinosuchus* and *Purussaurus* that reached estimated lengths of up to nine metres (Erickson & Brochu, 1999; Paiva et al., 2022). Alligatoroids also had a broader past distribution than today, including inhabiting Europe (e.g. Martin et al., 2014) and the Arctic Circle (Estes & Hutchison, 1980), with early caimanines also present (and probably originating) in North America (Bona et al., 2018; Brochu, 2010; Cossette & Brochu, 2018). The anatomical and macroevolutionary history of this diversification and its subsequent decline is increasingly being constrained by fossil discoveries (e.g. Bona et al., 2018; Cossette & Brochu, 2018; Shan et al., 2021) and new analytical approaches (e.g. Fernandez Blanco et al., 2023; Godoy, 2020; Iijima et al., 2018; Paiva et al., 2024). Less clear is how life history and thermophysiology within alligatoroids might have changed, especially given the contrast between their past and present‐day ecomorphological diversity and spatial distribution (Erickson & Brochu, 1999).

A key tool to reconstructing physiological processes, especially in extinct taxa, is osteohistology. As bone provides a record of growth (Amprino, 1947; de Ricqlès, 1976), metabolism (Castanet et al., 2000; Padian & Horner, 2004), and ontogenetic responses to changing biomechanical demands (Cowin, 1990; Wolff, 1893) in extant vertebrates, osteohistology presents a unique opportunity to reconstruct the life history of extinct species (de Buffrénil, Quilhac, et al., 2021; Lamm, 2013). Osteohistological data of extant crocodylians is primarily based on *Alligator mississippiensis* (Klein et al., 2009; Lee, 2004; Rainwater et al., 2022; Tumarkin‐Deratzian et al., 2007; Woodward et al., 2011, 2014), which is commonly used as a model organism when discussing archosaurian growth in the fossil record (e.g. de Ricqlès et al., 2003; Hoffman et al., 2019; Scheyer, 2024; Scheyer & Desojo, 2011). From these data, a number of interpretations about crocodylian physiology have been drawn, such as supporting determinate growth as the primary pattern (Rainwater et al., 2022; Tumarkin‐Deratzian et al., 2007; Woodward et al., 2011), a strong, seasonally‐controlled cyclicity in this growth (Woodward et al., 2014), and relatively faster growth early in ontogeny (Chinsamy & Hillenius, 2004; Tumarkin‐Deratzian et al., 2007; Woodward et al., 2014). Recent osteohistological work on the extant caimans *Caiman yacare* (Andrade et al., 2020) and *Caiman latirostris* (Mascarenhas‐Junior et al., 2021; Pereyra et al., 2024), has demonstrated that these growth patterns characterize living alligatoroids more broadly. Data from recapture studies have demonstrated that living alligatoroid species grow at similar overall rates to one another, but smaller‐bodied taxa reach skeletal maturity at an earlier age, evidenced by asymptotic growth (Campos et al., 2014; Da Silveira et al., 2013; Wilkinson et al., 2016).

Given that extant alligators and caimans exhibit similar physiologies and osteohistological signatures, it is most parsimonious to conclude that these growth patterns were also present in early alligatoroids. However, given the much greater ecomorphological diversity of extinct alligatoroids than those of today, and the long divergence time (>60 Ma) for the alligator–caiman split (e.g. Roos et al., 2007), it is possible that similar growth patterns in extant alligators and caimans evolved convergently. For example, this might have been in response to the rapid climatic changes of the Pleistocene and Holocene (e.g. Seersholm et al., 2020). To test which of these two scenarios is likely to be correct, osteohistological studies of extinct alligatoroids are needed. However, such studies are scarce (e.g. Erickson & Brochu, 1999; Andrade et al., 2020; de Buffrénil et al., 2015; 2021). Furthermore, those of early‐diverging taxa (*Deinosuchus* sp., *Diplocynodon remensis*, and *Allognathosuchus wartheni*) have only been based on osteoderm sections (de Buffrénil et al., 2015; Erickson & Brochu, 1999), which have been demonstrated to be among the least informative elements in extant crocodylians for skeletochronology (i.e., determining age from growth marks), given that they are subject to disproportionate remodelling (e.g. Woodward et al., 2014). In addition, there are few opportunities in the fossil record to sample multiple individuals of the same crocodylian species, meaning that physiological reconstructions based on osteohistology might be affected by the inability to account for intraspecific variation. Features such as the presence/absence of bone tissue types and amount of vascularization are commonly used in thermophysiological and growth reconstructions, yet are clearly intraskeletally and ontogenetically variable in studies of extant species (Pereyra et al., 2024; Woodward et al., 2011, 2014). This complicates reconstructions of the evolution of growth and metabolism in alligatoroids, obscuring whether alligators and caimans share a plesiomorphic condition or convergently evolved similar growth patterns.

Here, we evaluate the osteohistology of the early‐diverging alligatoroid *Diplocynodon hantoniensis* from the late Eocene of the UK (Rio et al., 2020; Wood, 1846). We sample nine individuals representing an ontogenetic series from the same locality and stratigraphic horizon. Specifically, we document shifts in microanatomical and osteohistological features across a range of body sizes and ontogenetic stages, as well as quantitatively reconstruct allometric patterns and growth curves. These data are then compared with extant alligatoroids, providing a unique window into growth dynamics and life history evolution of an extinct, early‐diverging species of this group.

## 2 MATERIALS AND METHODS

Nine femora assigned to *Diplocynodon hantoniensis* (Rio et al., 2020) were selected for study, capturing the size disparity in available specimens (Table 1). These femora are accessioned in the Natural History Museum, London, United Kingdom (NHMUK). All specimens are from the type locality and horizon of *Diplocynodon hantoniensis* at Hordwell Cliff, near Lymington, Hampshire, United Kingdom, emanating from the Totland Bay Member of the Headon Hill Formation (Rio et al., 2020), which is dated to the Priabonian (late Eocene) (Daley, 1999; Edwards & Daley, 1997). Given the size differences between specimens, we consider each specimen to represent a separate individual.

**TABLE 1:** Measurements of studied femora of *Diplocynodon hantoniensis*.

Specimen	FL	FMC	FTR	FPMn	FPMx	FDH	FDW	TL	S	P
PV OR 25231	147*	53	56	19	34	‐	‐	N/A	0.137	0.318
PV OR 25238	103*	57	22*	‐	‐	‐	‐	N/A	0.083*	0.318*
PV OR 30210	212	76	80	30	49	35	48	3079.9	0.060	0.353
PV OR 30222	109	33	37	10	23	16	21	1591.5	0.073	0.468
PV OR 30223	84	26	27	9	19	10	‐	1230.3	0.028	0.409
PV OR 30247	95*	41	31*	‐	‐	‐	‐	N/A	0.028	0.516
PV OR 30399	205	79	74	26	47	35	‐	2978.7	0.107	0.354
PV R 5215	128	42	43	13	29	18	28	1866.1	0.049	0.409
PV R 5267	136*	65	66	25	45	‐	‐	N/A	0.043	0.401

*Note*: Asterisks indicate a measurement based on an incomplete element and dashes indicate an absent measurement. All measurements are in millimetres. Calculated using Farlow et al. (2005); S, 1/slope of the sigmoid from BoneProfileR parameters; P, distance of the inflexion point to the bone center from BoneProfileR parameters.

Abbreviations: FL, femoral length; FMC, femoral midshaft circumference; FTR, distance from proximal articular end of bone to fourth trochanter; FPMn, femoral proximal head minimum length; FPMx, femoral proximal head maximum length; FDH, femoral distal head height; FDW, femoral distal head width; TL, total length.

Osteohistological sections were made transversely at the femoral midshaft. All sections were prepared at the NHMUK following best practice protocols (Cerda et al., 2020; Lamm, 2013). Sections were analysed using a Leica DM750P microscope under plane‐ and cross‐polarized light. Photographs of osteohistological slides were taken using an Axio Imager M2, with an AxioCam HR RS camera, at the NHMUK. High‐resolution images of each section in plane‐ and cross‐polarized light are available on Morphosource (Project ID: 000694161).

**FIGURE 1:**
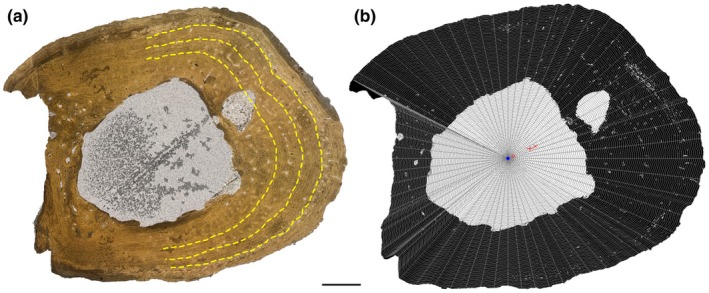
Cross section of the femoral midshaft of *Diplocynodon hantoniensis* PV OR 30223. (a) Cross section in PPL. Yellow dashed lines illustrate half‐circumference measurements. (b) Same cross section converted to a black and white image for compactness analysis in BoneProfileR. Radial lines illustrate how the section was divided up for calculation of compactness, using the ontogenetic centre (blue circle) as starting point. x, centre of mineralized part; o, centre of non‐mineralized part; +, centre of the section.

We use the terminology presented by de Buffrénil and Quilhac (2021) and de Buffrénil, Amson, et al. (2021) for qualitative descriptions of osteohistological and microanatomical features of the sections. Quantitative measurements of these features were captured using ImageJ, v. 1.54d (Abràmoff et al., 2004, https://imagej.net/ij/, RRID:SCR_003070). Cyclical growth marks (CGMs) were numbered and counted, beginning nearest the medullary cavity and proceeding towards the outer surface. In most sections, tracing a CGM along its entire circumference was not possible because of a combination of remodelling and specimen damage, so we instead traced it along half the circumference in a subset of specimens where this was possible (Figure 1(a)), modified from the complete circumferences of Woodward et al. (2014). Additionally, the linear distances between CGMs were measured as a record of growth, as these are more reliably preserved than circumferences. These distances were taken from four measurements along the major axes from the geometric centroid to each successive CGM and then averaged to provide an annual periosteal thickness, though in some cases a sequence could only be measured in two or three directions. In both cases, the limit imposed by remodelling means the first measurable CGM is likely not reflective of the chronologically oldest age for the individual. As such, we present data as from the first CGM, rather than assign age, following previous studies (e.g. Woodward et al., 2014). Quantitative inference of ecological habit, e.g. marine, semiaquatic, terrestrial, is possible using bone compactness metrics on thin sections (Gônet et al., 2022). The distribution of bone compactness along the sections (Figure 1(b)) was quantified using the R v.4.3.1 1 (http://www.r‐project.org/, RRID:SCR_001905) package ‘BoneProfileR’ v.3.1 (Gônet et al., 2022), specifically parameters P (relative distance from the centre to the most abrupt change in compactness) and S (the width of the transition zone between medullary cavity/spongiosa to cortex). Osteohistological measurements of CGM half‐circumferences and distances between consecutive CGMs are presented in Supplementary Data.

Femoral length and midshaft circumference measurements (Table 1) were taken with mechanical callipers prior to sectioning and follow the definitions of Farlow et al. (2005) and Iijima and Kubo (2019). We used the formulae from Farlow et al. (2005) to estimate total body length for *D. hantoniensis* primarily from femoral length, although there is some uncertainty when using a single skeletal element for size estimates in crocodylians (Iijima et al., 2018). For allometric analysis of femoral midshaft circumferences against femoral length, all measurements were log_10_‐transformed before conducting reduced major axis (RMA) regressions using the R v.4.3.1 (http://www.r‐project.org/, RRID:SCR_001905) package ‘smatr’ v.3.4–8 (Warton et al., 2012), following the methodology of Iijima and Kubo (2019). We used the slope.test function in ‘smatr’ to statistically test whether the RMA slopes differ from isometry. The relationship between femoral midshaft circumference and length was considered to be positively/negatively allometric when the RMA slopes' 95% confidence intervals exclude isometry (slope = 1).

## 3 RESULTS

### 3.1 Osteohistological and microanatomical description

**FIGURE 2:**
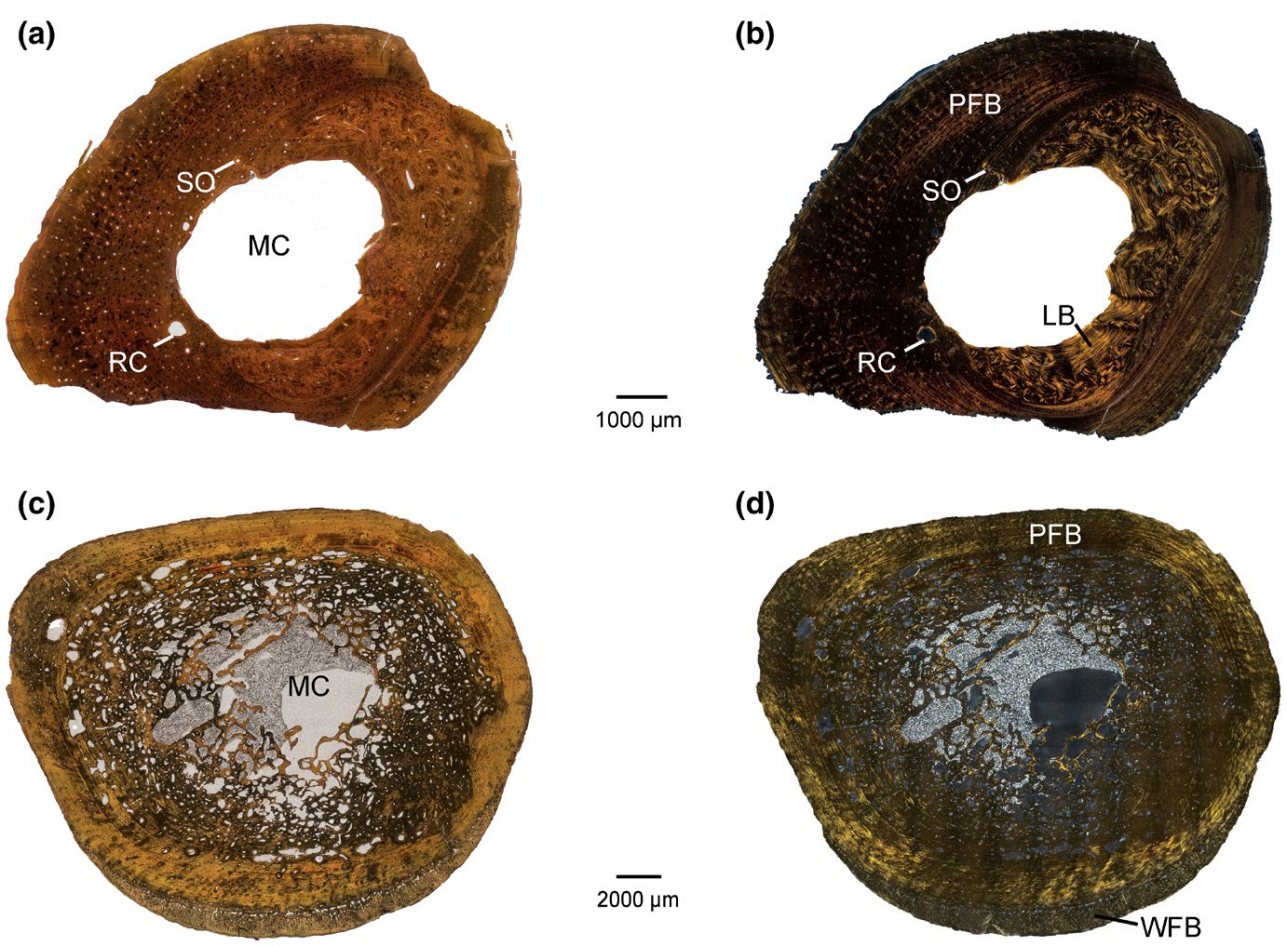
*Diplocynodon hantoniensis* femoral mid‐shaft sections. (a) PV OR 30222 in PPL (b) PV OR 30222 in XPL (c) PV OR 30399 in PPL (d) PV OR 30399 in XPL. LB, lamellar bone; MC, medullary cavity; PFB, parallel‐fibred bone; RC, resorption cavity; SO, secondary osteon; WFB, woven‐fibred bone.

**FIGURE 3:**
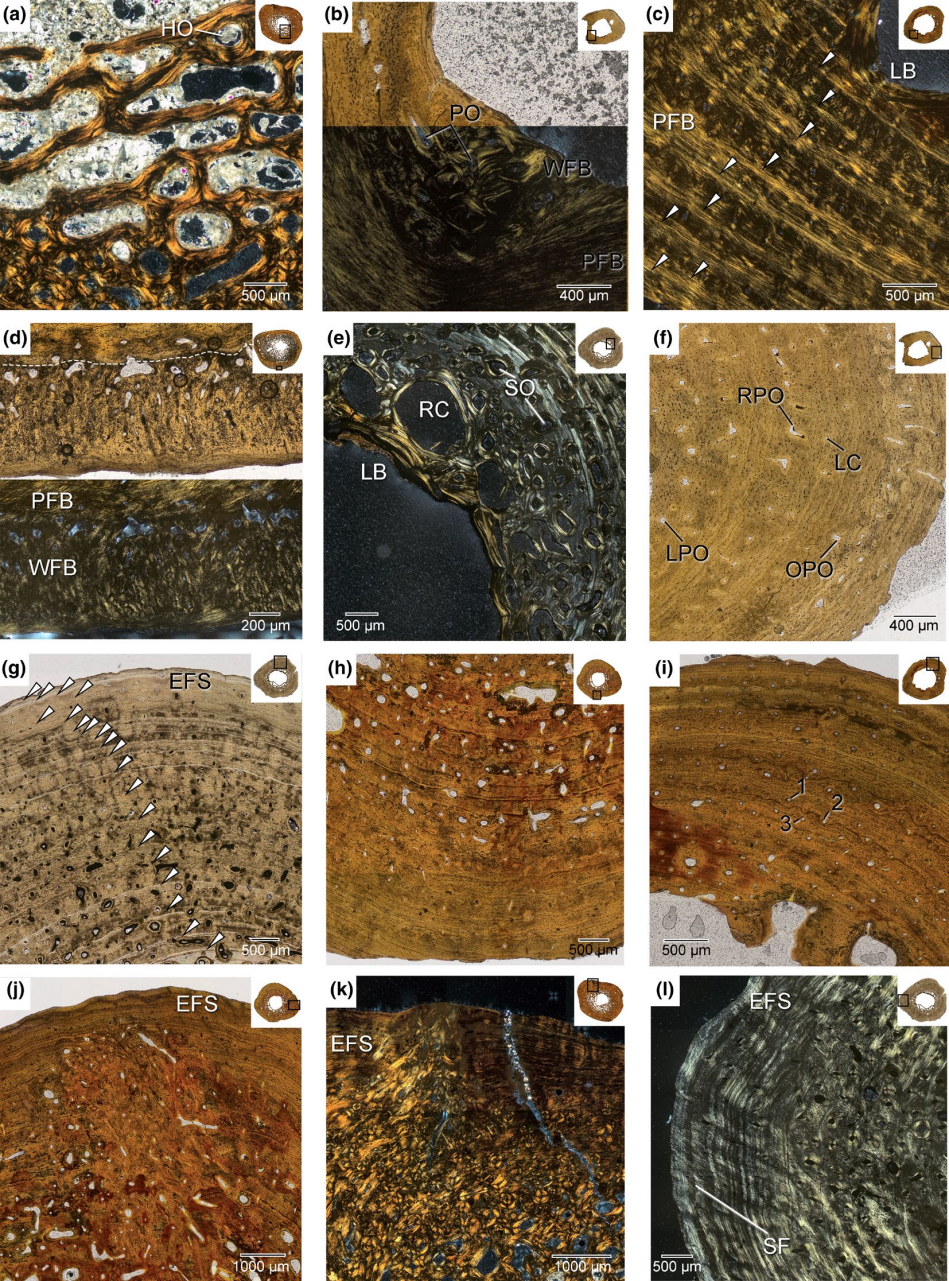
*Diplocynodon hantoniensis* femoral mid‐shaft sections in detail. (a) PV OR 25231 in cross‐polarized light (b) PV OR 30223 in plane‐ and cross‐polarized light (c) PV OR 30247 in cross‐polarized light (d) PV OR 30399 in cross‐polarized light (e) PV R 5267 in cross‐polarized light (f) PV OR 30223 in plane‐polarized light (g) PV R 5267 in plane‐polarized light (h) PV OR 30210 PV R 5267 in plane‐polarized light (i) PV OR 30247 in plane‐polarized light. Numbers (1, 2, and 3) refer to the three stages of primary osteon formation as defined by de Buffrénil and Quilhac (2021) (j) PV OR 30210 PV R 5267 in plane‐polarized light (k) PV OR 25231 in cross‐polarized light (l) PV R 5267 in cross‐polarized light. White arrowheads indicate growth marks. EFS, external fundamental system; HO, hemi‐osteon; LB, lamellar bone; LC, longitudinal canal; LPO, longitudinal primary osteon; OPO, oblique primary osteon; PFB, parallel‐fibred bone; PO, primary osteon; RC, resorption cavity; RPO, reticular primary osteon; SF, Sharpey's fibres; SO, secondary osteons; WFB, woven‐fibred bone.

Given the general similarity among all specimens, we describe them all together, noting any variation. All sections reveal a well‐defined medullary cavity with endosteal bone enclosed by an external layer of compact periosteal bone (Figure 2). This periosteal bone comprises roughly the outer half of the cross‐section (Table 1, Figure 2(a)), although periosteal bone forms only the outer‐most third of the diaphysis in some of the largest femora (e.g. NHMUK PV OR 25231 and PV OR 30999) due to erosion cavities and subsequent replacement by endosteal bone (Table 1, Figure 2(c)). This results in an abrupt change in bone compactness from the medullary cavity to cortex, captured quantitatively by low values of S, indicating a distinct compact cortex and a medullary cavity (Table 1). In all sections except NHMUK PV R 5267, the endosteal bone is less dense in the posterior regions in comparison to the anterior, lateral, or medial regions, with greater intertrabecular spaces, although the same thickness (Figure 2(c),(d)). NHMUK PV R 5267 preserves a slightly denser posterior region than the remainder of the section. For the most part, trabeculae are randomly orientated, but are more parallel and circumferential toward the central part of the section in all quadrants (Figure 3(a)). There are numerous secondary osteons and hemi‐osteons (i.e. structurally equivalent to secondary osteons, except located on trabeculae as the result of trabecular remodelling, sensu de Buffrénil and Quilhac (2021)) (Figure 3(a)). In the larger and presumably older individuals (i.e. NHMUK PV OR 25231, PV OR 25238, and PV OR 30999), remodelling is more extensive, with a wider spongious transition zone between the medullary cavity and the compact cortex. In the smallest individuals (NHMUK PV OR 30222 and OR 30223), resorption is restricted to the anterolateral half of the section and secondary deposition on the posteromedial half of the section. This is indicative of cortical drift, as most clearly evident in NHMUK PV OR 30222, wherein the thickness is the same medially and laterally, but the medial wall is composed of approximately 50% deposited endosteal lamellar tissue, whereas the lateral wall consists of primary parallel‐fibred bone (PFB) of periosteal origin (often referred to in older literature as ‘lamellar‐zonal’ tissue; see de Buffrénil & Quilhac, 2021) (Figure 2). This directionality is less apparent in larger specimens, in which remodelling occurs throughout the section, obscuring evidence of drift. The S parameter of bone compactness (Table 1) is indicative of tubular bones (*S* = 0.028–0.137), with an abrupt transition from medullary cavity to compact periosteal bone. These values highlighting a distinct compact cortex and the general bone compactness profiles (P = 0.318–0.516) indicate a semiaquatic habit for *D. hantoniensis*, based on comparisons with the bone compactness of other crocodylians (P = 0.232–0.694 and S = 0.015–0.081; de Buffrénil et al., 2021b).

In all specimens, the cortex is predominately composed of parallel‐fibred bone (PFB). The PFB can be identified by its anisotropy, showing second‐order birefringence and entering extinction uniformly in cross polarized light, as well as by osteocyte lacunae that are elongate and parallel to the direction of bone deposition (Figure 3(c)). This PFB is well vascularized (see subsequent paragraph for details) and shows numerous growth marks (annuli and LAGs). In all cases, the osteon and lacunae density of the PFB decreases from the medullary cavity to the external surface. The only occurrences of woven‐fibred bone (WFB) are as pathological tissue growth on the medial periosteal surface of NHMUK PV OR 30399 (Figure 3(d)), in an instance of abnormal growth that is clearly visible macroscopically on the femoral shaft, and as primary periosteal tissue internal to the first LAG of the smallest specimen, NHMUK PV OR 30223 (Figure 3(b)). WFB can be identified in cross polarized light by its isotropy and in plain polarized light by the presence of large, multipolar, and randomly oriented osteocyte lacunae (Figure 3(b)). Lamellar bone (LB) is solely present endosteally in relation to cortical drift (Figure 2(b)). The LB can be differentiated from PFB by the presence of lamellae and an alternate reaction pattern between monorefringence and birefringence successive lamellae in polarized light; LB is limited to filling secondary osteons, as well as endosteal bone that are associated with remodelling (Figure 3(e)). Additionally, NHMUK PV OR 30222 preserves a large patch of compacted coarse cancellous bone (CCCB) along the medial and posterior portions of the medullary cavity (Figure 2(a),(b)). CCCB is most commonly restricted to the metaphyseal region, it can migrate to the diaphyseal region (Enlow, 1963), including in the diasphyses of pseudosuchians (de Ricqlès et al., 2008). The significance of CCCB in the diaphysis is unclear, with recent work on mammal osteohistology suggesting a biomechanical purpose (e.g. Heck et al., 2019; Legendre & Botha‐Brink, 2018), although the authors of both studies noted that more investigation is needed to understand CCCB's functional role.

Vascularization is composed of simple canals and mostly narrow (ca. 50–80 μm) primary osteons (in smaller samples like NHMUK PV OR 30223 primary osteons can reach 300 μm or 100–150 μm in WFB and PFB, respectively). Vascular openings are primarily longitudinal, with cases of obliquely oriented canals and primary osteons and reticular anastomoses throughout the range of size classes, although in larger specimens the relative amount of reticular and oblique openings decreases (Figure 3(f–i)). The degree of vascularization and orientation of vascular canals and osteons varies within individual sections and across the size gradient of sections, as in other crocodylians (de Buffrénil et al., 2021b): vascular density decreases towards the outer cortex in all sections and larger individuals have lower overall vascular density than smaller individuals (Figure 3(f–i)). This is best demonstrated in NHMUK PV OR 30247 (Figure 3(i)), which also preserves all stages of primary osteon formation (see de Buffrénil, Amson, et al., 2021), along with preserving all the types of simple canals and primary osteons that occur in our sample (Figure 3(i)). These vascular openings occur within zones of PFB, although larger primary osteons (ca. 100–300 μm) also occur in the pathological WFB in NHMUK PV OR 30399 and in the non‐pathological WFB in NHMUK PV OR 30223 (Figure 3(b),(d)). In areas with WFB, primary osteons are oriented longitudinally and in reticular anastomoses: they are evenly distributed in the primary tissue of NHMUK PV OR 30223 (Figure 3(b)) and concentrated on the boundary between the pathological tissue and periosteal tissue in NHMUK PV OR 30399 (Figure 3(d)).

As is the case with vascularity, the density of osteocyte lacunae decreases towards the external surface, and they tend to become flatter along the axis perpendicular to growth towards the external surface. Sharpey's fibres (SF) are present in all sections. All but the smallest specimen (NHMUK PV OR 30223) preserve SF with higher frequency in the posterior portions of the sections. In the case of NHMUK PV OR 30223, the posterior region is largely missing, but there are SF in less dense concentrations throughout the section. In three of the specimens (NHMUK PV OR 25231, PV OR 30210, PV R 5215), there is a patch of PFB located in the posteromedial portion of the section with tissue fibres that are not oriented parallel to the adjacent tissue fibres and instead follow the orientation of the SF (Figure 3(j),(k)). This region is also more vascularized than tissue elsewhere in the cortex (Figure 3(j),(k)). This patch is located beneath a muscle scar visible on the bone surface just distal to the fourth trochanter. Based on this location, the muscle scar could be the insertion for the pubo‐ischio‐femoralis internus or caudofemoralis longus and brevis, or the origin for the femorotibialis externus (Allen et al., 2010; Hutchinson, 2001; Maidment & Barrett, 2011).

### 3.2 Growth indicators

**FIGURE 4:**
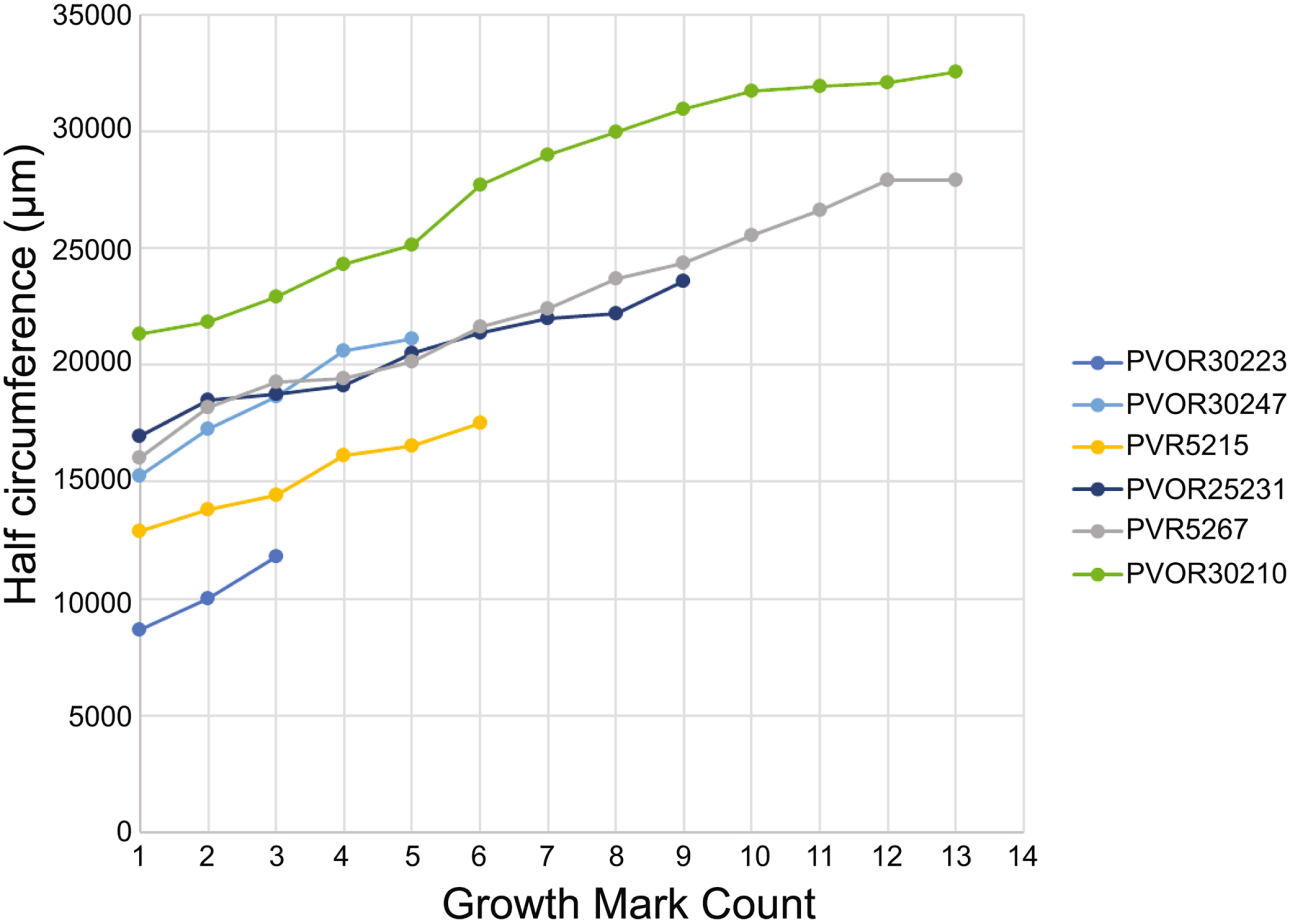
Half circumference of cyclical growth marks (CGMs) of *Diplocynodon hantoniensis* in μm. These data provide a linear record of annual growth for the subset of samples with CGMs that are traceable over at least half the circumference. As growth marks can be lost through ontogeny and non‐annual factors can result in the preservation of a CGM, it is more appropriate to plot counts starting at the first measurable CGM rather than numerical age, with the further caveat that the first CGM is not directly comparable between individuals. Rather, the overall pattern of growth recorded in all specimens indicates a fairly consistent, linear slope with a potential asymptote in the largest specimen, PV R 5267.

**FIGURE 5:**
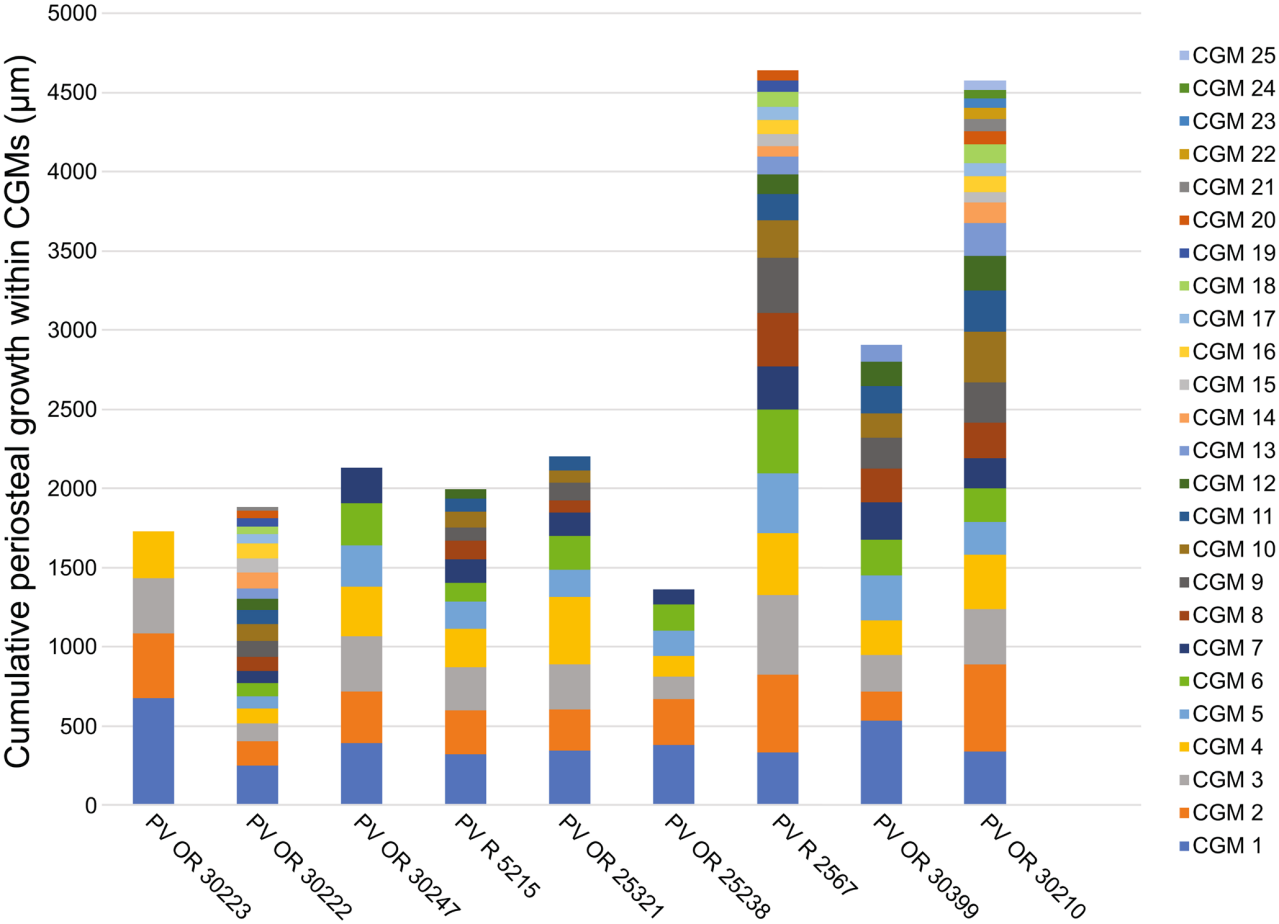
Annual periosteal radial thickness of *Diplocynodon hantoniensis* in μm. Averaged measurements were taken along the major axes from the geometric centroid to each successive CGM to provide an annual periosteal thickness. As with circumferential measurements growth mark counts start at the first measurable CGM rather than numerical age, with the further caveat that the first CGM is not directly comparable between individuals.

There are numerous CGMs in all sections of *D. hantoniensis*, including both LAGs and annuli (Figure 3(c),(g)). The type of CGMs present (LAGs or annuli) vary by individual and year, rather than reflecting tissue types or body size. Some sections record only annuli (e.g. NHMUK PV OR 30223) or a mix of LAGs and annuli (e.g. NHMUK PV OR 30222). The high degree of remodelling in most sections results in incomplete counts of CGMs, complicated by some sections being damaged (most notably NHMUK PV OR 25238). Even with this limitation, the sample includes a range of CGMs from four in NHMUK PV OR 30223 (the smallest specimen) to 25 in NHMUK PV OR 30210 (the largest specimen). Surprisingly, the second smallest specimen (NHMUK PV OR 30222) possesses the second highest CGM count with 21 CGMs. In five of the nine specimens (NHMUK PV OR 25231, PV OR 30210, PV OR 30222, PV OR 30999, and PV R 5267), the CGMs are closely spaced near the outer margin of the cortex, indicative of an external fundamental system (EFS). Growth rates appear to be consistent with a similar slope across specimens, although the largest of these specimens (NHMUK PV R 5267) shows a plateauing at the end which is reflected by the formation of an EFS (Figure 4). Annual (see Discussion for caveats) periosteal growth, measured as the distance between consecutive CGMs, was more readily available than circumferential measurements and, when plotted, shows decelerating growth, with CGM count reaching an asymptote (Figure 5). However, there is variation within individuals, occasionally resulting in increased amounts of deposition (Figure 5). There is no evidence of embryonic bone in any of the *D. hantoniensis* sections, which is unsurprising given that these tissues are only preserved in the youngest (6 months to 1 year) individuals of *Caiman latirostris*, the only alligatoroid taxon for which they have been described (Pereyra et al., 2024). Using the methods of Farlow et al. (2005), we calculated total body lengths ranging from 1.2–3.4 m in our sample (Table 1). Compared to *Alligator mississippiensis*, these values are typical of a range of skeletally immature subadults to fully mature adults, congruent with histological data from our sample (Wilkinson et al., 2016).

### 3.3 Allometry

**FIGURE 6:**
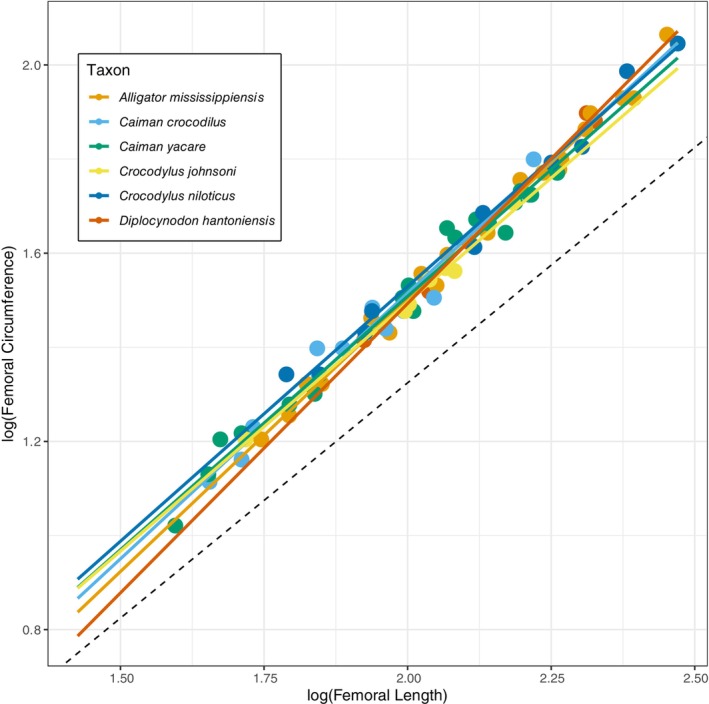
Log–log reduced major axis (RMA) regressions between crocodylian femoral lengths and midshaft circumferences. Dashed line is the isometric slope (=1) with y‐intercept from whole sample regression using all available individuals.

**FIGURE 7:**
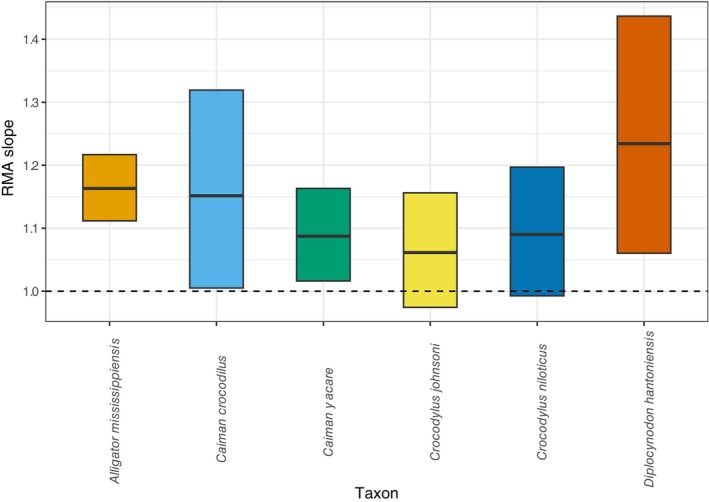
Comparison of reduced major axis (RMA) slopes for crocodylian femoral lengths and midshaft circumferences. Boxplots show RMA slope means (plotted in Figure 6) and their 95% confidence intervals. Dashed line is the isometric slope (=1).

The femoral midshaft circumference is positively allometric with respect to femoral length in *D. hantoniensis* (slope = 1.23, *R* ^2^ = 0.93, *p* = 0.021; Figure 6). The slope confidence interval of *D. hantoniensis* (1.06–1.44) is similar to those of *Alligator mississippiensis*, *Caiman yacare*, and *Caiman crocodilus* (Iijima & Kubo, 2019), but differs from those of *Crocodylus johnstoni* and *Crocodylus niloticus*, which exhibit isometric growth (Iijima & Kubo, 2019) (Figure 7).

## 4 DISCUSSION

This study provides the first comprehensive assessment of growth and histovariability in a fossil alligatoroid. Below, we discuss intraspecific variation in the osteohistology of *Diplocynodon hantoniensis*. The most ontogenetically comprehensive osteohistological studies of extant alligatoroids are of *Alligator mississippiensis* (Woodward et al., 2014) and *Caiman latirostris* (Mascarenhas‐Junior et al., 2021; Pereyra et al., 2024), so our comparisons below primarily focus on these two species.

### 4.1 Osteohistology and microanatomy

As is the case in extant crocodylians more generally, the internal bone microanatomy of *D*. *hantoniensis* is characterised by relatively simple vascularisation when compared to mammals and dinosaurs (Cubo et al., 2020; de Buffrénil et al., 2021b). Like extant crocodylians (e.g. de Buffrénil et al., 2021b; de Ricqlès et al., 2003; Woodward et al., 2014), *D*. *hantoniensis* produced WFB within the first CGM as in NHMUK PV OR 30223. Interestingly, WFB is also preserved as a pathological tissue in NHMUK PV OR 30399 which represents a pathological growth on the surface of the femur clearly visible externally. This demonstrates *D. hantoniensis* was capable of situationally depositing WFB as a skeletally mature adult, at least locally. All sampled sections of *D. hantoniensis* are dominated by PFB, which is the dominant tissue type in all sampled extant crocodylians (de Buffrénil et al., 2021b and references therein). As in *Alligator mississippiensis* and *Caiman latirostris*, *D. hantoniensis* underwent numerous changes in histological and microanatomical features through ontogeny. Larger femora contain less dense and simpler vascularization in the outermost zones and, within the same section, the degree of vascularization decreases towards the outer cortex, indicating a decelerating growth rate. Quantitative measures, such as the compactness distribution parameters P and S, vary across our sample and even exceed the variation presented in taxonomically diverse studies of crocodylomorphs (e.g. de Buffrénil et al., 2021b). For example, the range of S values in our sample equals 0.109, whereas the range across 11 crocodylomorph taxa of a similar size range in a recent study equals 0.066 (de Buffrénil et al., 2021b). This variation does not correlate with size, as both the smallest (NHMUK PV OR 30222) and largest (NHMUK PV OR 30210) individuals in our sample have parameter values in the middle of the range (Table 1). Vascularization and bone compactness measures are important for thermophysiology and habitat reconstructions, respectively, and our results indicate that these are susceptible to ontogenetic and individual variation. Our results highlight the importance of specimen selection and a need for awareness of limitations of applying results of singleton histological analyses when interpreting life histories of extinct animals.

### 4.2 Growth

Similar to *Alligator mississippiensis*, the type of CGM (LAG vs. annulus) varies within and between individuals in *D. hantoniensis*, rather than being dependent on tissue arrangement or ontogenetic stage (Woodward et al., 2014). As in all extant crocodylians, the periosteal tissue of *D. hantoniensis* is dominated by ‘lamellar‐zonal’ tissue, a type of tissue composed of parallel‐fibred bone arranged in what are assumed to be annual zones and annuli (see de Buffrénil & Quilhac, 2021 for additional discussion). An important caveat in osteohistological reconstructions of growth is the assumption that all CGMs represent an annual cessation of growth and that this annual cessation is recorded. Recent work has demonstrated that skeletochronological data are also influenced by non‐annual events, such as starvation or disease (Schucht et al., 2021), and deposition can be complicated by double/multiple CGMs which can split and merge across the circumference (Cullen et al., 2021; Schucht et al., 2021). Contrastingly, D'Emic et al. (2023) argued that these inconsistencies in CGMs was the result of methodological error and found CGMs do reflect accurate ages.

If the CGMs of *D. hantoniensis* represent an accurate record of yearly growth, then the sample ranges from ages of 5 to 26 with most the sample over 10. This is consistent with the majority of the specimens showing evidence for skeletal maturity (i.e. EFS present) and extensive remodelling. Living alligatoroids reach maximum body sizes at between 12 to 18 in caimans (Campos et al., 2014; Da Silveira et al., 2013) and 30 to 40 in *Alligator mississippiensis* (Wilkinson et al., 2016). Given the CGM counts of our specimens represent minimum age estimates, we find *D. hantoniensis* reached asymptotic growth, and therefore maximum bod size at ages consistent with that of living alligatoroids. Additionally, CGMs are useful for reconstructing growth dynamics and stage of skeletal maturity; furthermore, sampling multiple individuals enables some control over random events that might complicate the growth record of a single individual (e.g. Curry Rogers et al., 2024; de Buffrénil & Castanet, 2000; Pereyra et al., 2024).

In vertebrates with determinate growth, including extant crocodylians, such as *A*. *mississippiensis* (Klein et al., 2009; Werning, 2013), *Caiman latirostris* (Pereyra et al., 2024), and *Crocodylus niloticus* (Chinsamy, 1991), the spacing of growth marks decreases from the inner to the outer cortex and results in the formation of an EFS. Five specimens of *D. hantoniensis* show evidence of an EFS (Figure 3). Surprisingly, one of these sections is from an individual (PV OR 30222) that is approximately 50% the femoral length and/or femoral midshaft circumference of the other specimens with an EFS. In extant alligatoroids, females reach asymptotic growth at both smaller sizes (approximately 25%–50% snout‐vent length) and five to ten years younger than males of the same species (Campos et al., 2014; Da Silveira et al., 2013; Wilkinson et al., 2016). It is possible that such sexual dimorphism existed within *D. hantoniensis* as well, although demonstrating this in the fossil record is difficult (e.g. Hone et al., 2020). Alternatively, this could represent individuals growing during/through different environmental conditions (e.g. de Buffrénil & Castanet, 2000), as these individuals were likely preserved over an extended period of time, although studies of extant alligatoroids have found only a weak link between environmental factors and growth (Campos et al., 2014; Da Silveira et al., 2013) with growth being primarily controlled by annual seasons (Andrade et al., 2020; Campos et al., 2014). There is histological evidence for developmental plasticity in crocodylomorphs (e.g. Spiekman et al., 2024); however, those studies also suggested alternative interpretations, such as intraskeletal variation (Spiekman et al., 2024). Here, we find evidence of asymptotic growth in *D. hantoniensis* based on the presence of an EFS, as well as in our quantitative plots (Figures 4, 5), that is consistent with the findings of determinate growth in extant alligatoroids (Rainwater et al., 2022; Tumarkin‐Deratzian et al., 2007; Woodward et al., 2011). Importantly, this contrasts with existing data on extinct alligatoroid growth, which indicated non‐asymptotic growth in the giant early‐diverging alligatoroid *Deinosuchus* (Erickson & Brochu, 1999). Our findings suggest that there might be greater complexity in early alligatoroid growth strategies than is present in living alligatoroids, but that the extant condition was established early in the clade.

### 4.3 Allometry

Femoral circumference scales positively with femoral length in *D. hantoniensis*, as is the case in the extant alligatoroids *Alligator mississippiensis*, *Caiman crocodilus*, and *Caiman yacare* (Iijima & Kubo, 2019). However, this pattern does not hold more broadly across Crocodylia; for example, *Crocodylus johnstoni* and *Crocodylus niloticus* both exhibit an isometric relationship (Iijima & Kubo, 2019) (Figure 7). This lends support for similar biomechanical loading regimes among *D. hantoniensis* and extant alligatoroids, as the relationship between stylopodial circumference and length is correlated with an element's resistance to torsion, bending, and compression (Blob, 2000; Iijima & Kubo, 2019). However, because these extant taxa inhabit a wide range of lowland environments, including forest, rivers, and wetlands (Campos et al., 2010; Elsey & Woodward, 2010), previous work has been unable to link this shared biomechanical loading to habitat preference or the amount of terrestrial locomotor activity (Iijima & Kubo, 2019). Shared allometric trends in a clade are thought to be the result of either similar ecological demands across ontogeny or phylogenetic constraints on growth (Iijima & Kubo, 2019; Meers, 2002). Given the lack of ecological explanations for the shared relationships among alligatoroids, our results provide tentative evidence that it might therefore reflect a primarily phylogenetic, rather than an ecological, signal.

## 5 CONCLUSIONS

This study represents the largest osteohistological sampling of a single fossil crocodylian species to date, providing a unique window into intraspecific variation in an extinct species. Overall, the early‐diverging alligatoroid *Diplocynodon hantoniensis* shares many osteohistological features with living alligatoroids; this includes periosteal bone that is primarily composed of parallel‐fibred bone, as well as vascularization that is dominated by simple canals and primary osteons oriented longitudinally and obliquely. We find support for both determinate and seasonally controlled growth, as in extant alligatoroids, including *Alligator mississippiensis* and *Caiman latirostris* (Andrade et al., 2020; Pereyra et al., 2024; Woodward et al., 2011, 2014). Altogether, this demonstrates a strong conservation of the alligatoroid growth plan since at least the alligator–caiman divergence over 60 million years ago. Additionally, our allometric analysis indicates there might be phylogenetic constraints among alligatoroids established early in the clade. We also find support for potential sexual dimorphism in *D. hantoniensis*, related to maximum body size and age of skeletal maturity, that is possibly plesiomorphic for alligatoroids. Alternatively, such variation in growth and skeletal maturity could be the result of developmental plasticity.

Several features in our sample are only present in a single individual, such as the presence of primary WFB, or vary ontogenetically across the sample, such as the amount and orientation of vascularization. Both of these features are often used to reconstruct growth rates and infer thermophysiology in extinct taxa. Specimen limitations for extinct taxa mean that larger samples of a single taxon are not always possible, but our work demonstrates the importance of including individual exemplar taxa for capturing intraspecific variation within the context of clade‐wide studies.

## AUTHOR CONTRIBUTIONS

DKH contributed to concept/design, acquisition of data, data analysis/interpretation, drafting of the manuscript, critical revision of the manuscript and approval of the article. ERG contributed to data analysis/interpretation, critical revision of the manuscript and approval of the article. AH contributed to data analysis/interpretation, critical revision of the manuscript and approval of the article. SCRM contributed to acquisition of data, data analysis/interpretation, critical revision of the manuscript and approval of the article. RNF contributed to data analysis/interpretation, critical revision of the manuscript and approval of the article. PDM contributed to concept/design, data analysis/interpretation, critical revision of the manuscript and approval of the article.

## FUNDING INFORMATION

This work was supported by funding from NERC (NE/X014010/1), The Leverhulme Trust (RPG‐2021‐202), and The Royal Society (UF160216, URF\R\221,010).

## CONFLICT OF INTEREST STATEMENT

The authors declare no conflicts of interests.

## Supporting information


Data S1.

## Data Availability

The data that supports the findings of this study are available in the supplementary material of this article.
